# Integrated analysis of promoter methylation and expression of telomere related genes in breast cancer

**DOI:** 10.18632/oncotarget.16036

**Published:** 2017-03-09

**Authors:** Jianfu Heng, Fan Zhang, Xinwu Guo, Lili Tang, Limin Peng, Xipeng Luo, Xunxun Xu, Shouman Wang, Lizhong Dai, Jun Wang

**Affiliations:** ^1^ The State Key Laboratory of Medical Genetics & School of Life Sciences, Central South University, Changsha, Hunan, 410013, China; ^2^ Sanway Gene Technology Inc., Changsha, Hunan, 410205, China; ^3^ Department of Breast Surgery, Xiangya Hospital, Central South University, Changsha, Hunan, 410008, China; ^4^ Research Center for Technologies in Nucleic Acid-Based Diagnostics, Changsha, Hunan, 410205, China; ^5^ Research Center for Technologies in Nucleic Acid-Based Diagnostics and Therapeutics, Changsha, Hunan, 410205, China

**Keywords:** breast cancer, telomere, methylation, gene expression, biomarker

## Abstract

Telomeres at the ends of eukaryotic chromosomes play a critical role in tumorgenesis. Using microfluidic PCR and next-generation bisulfite sequencing technology, we investigated the promoter methylation of 29 telomere related genes in paired tumor and normal tissues from 184 breast cancer patients. The expression of significantly differentially methylated genes was quantified using qPCR method.

We observed that the average methylation level of the 29 telomere related genes was significant higher in tumor than that in normal tissues (*P* = 4.30E-21). A total of 4 genes (*RAD50, RTEL, TERC* and *TRF1*) showed significant hyper-methylation in breast tumor tissues. *RAD51D* showed significant methylation difference among the four breast cancer subtypes. The methylation of *TERC* showed significant association with ER status of breast cancer. The expression profiles of the 4 hyper-methylated genes showed significantly reduced expression in tumor tissues. The integration analysis of methylation and expression of these 4 genes showed a good performance in breast cancer prediction (AUC = 0.947).

Our results revealed the methylation pattern of telomere related genes in breast cancer and suggested a novel 4-gene panel might be a valuable biomarker for breast cancer diagnosis.

## INTRODUCTION

Breast cancer is the principal leading cause of cancer-related death among women worldwide [1]. In recent years, breast cancer has become the most frequently diagnosed cancer in Chinese women, accounted for 12.2% of global cases and 9.6% of related deaths from breast cancer worldwide [2]. Breast cancer is often diagnosed at the advanced stages due to nonspecific symptoms or lack of symptoms, leading to a poor prognosis [3]. The early diagnosis of breast cancer would improve the prospects of survival. Consequently, increasing studies have focused on the biomarkers for early diagnosis and new therapeutic targets for breast cancer.

Telomeres are caps of linear chromosomes at the chromosomal ends, which are protected by a number of molecules that constitute the capping shelterin complex [4, 5]. Cancer cells, characteristically acquire infinite capability to divide through maintenance of telomeres by sustained expression of telomerase, or by an alternative lengthening of telomeres (ALT) mechanism [6]. Telomere length maintenance is a complex process controlled by a large number of proteins including shelterin complexes, telomerase complexes and many DNA repair proteins [7]. The shelterin complex is consisted of six proteins including TRF1, TRF2, POT1, TIN2, TPP1 and RAP1, which packages telomeric DNA and protects the integrity and stability of chromosome during DNA replication [8]. Shelterin proteins interact with a number of other factors known as shelterin associated proteins that can influence integrity and dynamics of chromosome ends. These shelterin-associated proteins include TNKS1, TNKS2, and TEP [9, 10]. They are essential for the overall maintenance of genome integrity and prevent DNA degradation and chromosome end-to-end fusions [11]. Telomere dysfunction through telomere shortening and dysregulation of telomeric DNA-binding proteins has been reported in many kinds of cancers, including breast cancer [12]. It has been revealed that the telomere length was significantly associated with the risk and prognosis of breast cancer [13]. Telomerase is responsible for elongation of telomeric repeats at chromosomal ends and is important for controlling cell survival by maintaining telomere length [14]. Telomerase is a ribonucleoprotein enzyme composed of two essential components, a telomerase RNA template subunit and a catalytic protein subunit, telomerase reverse transcriptase (TERT) [15, 16]. These two subunits bind to H/ACA ribonucleoprotein complex containing dyskerin, NOP10, NHP2, and GAR1 which is necessary for synthesis and elongation of telomeric DNA [15]. It has been shown that the telomerase activity is silenced in almost all adult somatic cells but activated in more than 90% of cancers. Activation of telomerase is a fundamental step in tumorgenesis [9]. Genetic variation in *TERT*, *TRF1, TRF2, POT1, TEP, TNKS1, TNKS2, TP53, ATRX* and *DAXX* [17–24] and aberrant promoter methylation of *TERT*, *WRN*, *POT1, RAD50* and *TP53* [25–28] have been reported to contribute to the dysregulation of telomere length and telomerase activity in breast cancer.

Methylation within promoter regions serves as crucial regulator in tumorgenesis and has been suggested as a hallmark of cancers for its role in silencing gene expression [29–31]. Given their important functions in cancer initiation and progression, methylation changes have been considered as potential biomarkers for the early detection of cancers, including cervical, breast, bladder, gastrointestinal, and lung cancer [32–35]. However, the methylation patterns of most of the telomere related genes and their correlation with breast cancer are still unknown.

The purpose of the present study was to investigate the methylation of telomere related genes in breast cancer and identify new molecular biomarkers for breast cancer diagnosis and treatment. We analyzed 29 candidate genes in 184 breast cancer patients with high-throughput microfluidic PCR based target enrichment and next generation bisulfite sequencing method. The significantly differentially methylated genes were selected to analyze the correlation between promoter methylation and their expression. For the selected gene panel, further evaluation of its performance in breast cancer classification was implemented.

## RESULTS

### Methylation analysis of breast tumor and matched normal tissues

In the present study, methylation analysis of 29 telomere related genes was performed on 184 breast cancer patients with paired tumor and normal tissues using next generation bisulfite sequencing method. The MiSeq sequencing results showed that microfluidic PCR-generated libraries had highly sample and gene uniformity. About 90% of sequencing reads were mapped to the targeted promoter regions, and 97% of samples achieved coverage within 2-folds of the average reads.

**Figure 1:**
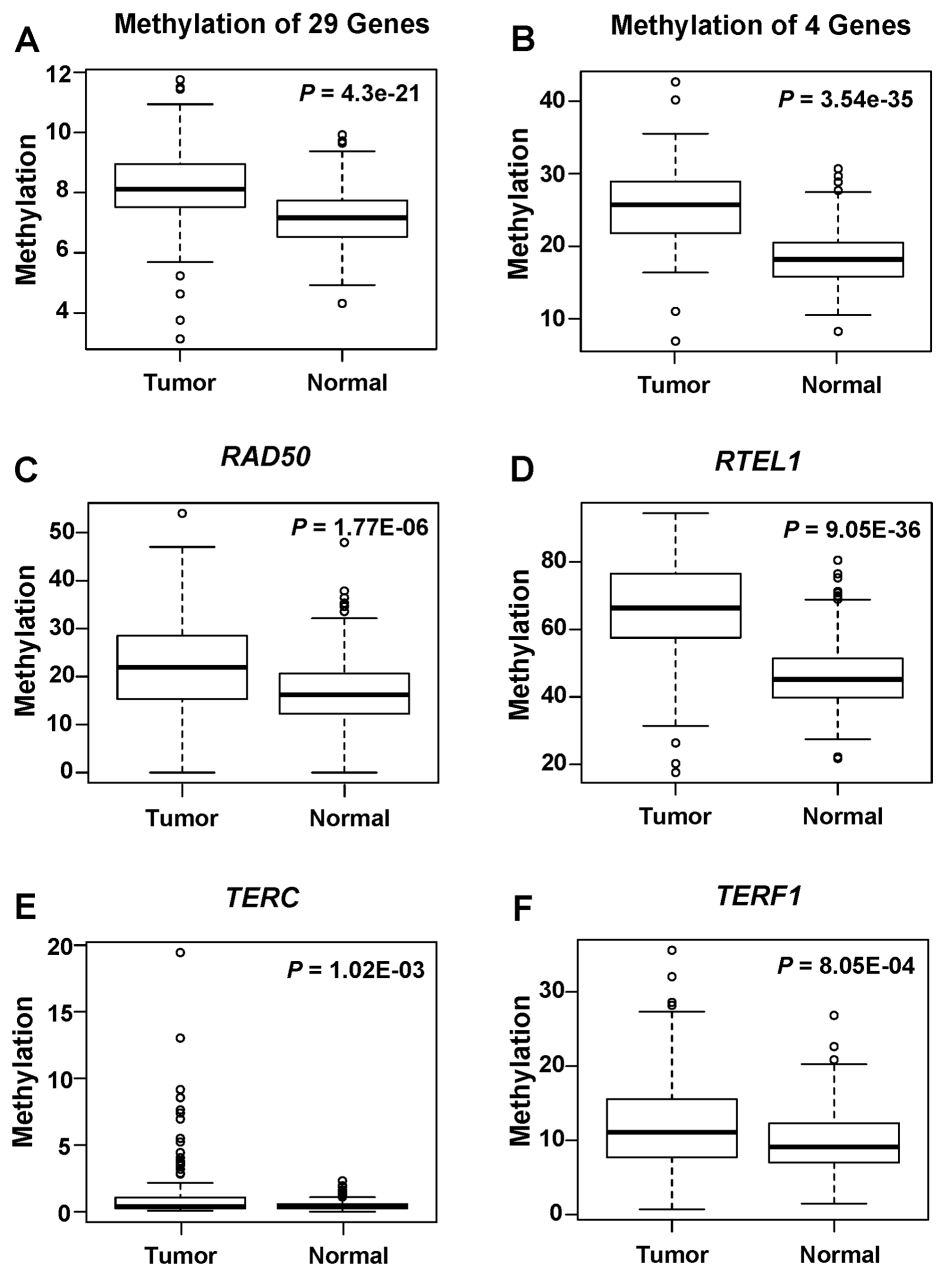
Boxplots for average methylation levels of candidate genes in 184 tumor and matched normal tissues The average methylation levels were shown for **(A)** 29 candidate genes, and **(B)** 4 hyper-methylated genes, respectively. *P* values were calculated using paired t-test. The average methylation levels were shown for **(C)** *RAD50*, **(D)** *RTEL*, **(E)** *TERC*, and **(F)** *TRF1* genes, respectively. *P* values were calculated using paired t-test and adjusted with Holm's correction procedure.

The average promoter methylation level of all candidate genes was summarized in Table 1. In general, the average methylation level of the 29 genes was 8.20% in tumor and 7.13% in normal tissue (*P* = 4.30E-21), and the average methylation level in 7 genes (*ATRX, DKC1, NBS1, RAD50, RAD51D, RTEL* and *TRF1*) was larger than 10%. It was obviously that the promoter methylation in most of the candidate genes was at a low level (< 1%). Paired t test revealed that 9 genes (*ATRX, NHP2, RAD50, RAD51D, RAP1, RTEL, TERC, TP53 and TRF1*) showed significant methylation difference between tumor and matched normal tissues. Except for *ATRX*, all of them showed hyper-methylation in breast cancer. The methylation difference of *RAD50, RTEL*, *TERC* and *TRF1* remained significant after Holm's correction (Table 1). The average methylation level of the 4 hyper-methylated genes showed highly significant difference between breast tumor and matched normal tissues (*P* = 3.54E-35) (Figure 1). Among them, *RTEL* showed the highest methylation level and the smallest *P* value for difference in methylation between breast tumor and normal tissues (corrected *P* = 9.05E-36) with close to 20% of methylation level change.

**Table 1:** The methylation level of 29 genes in tumor and normal tissues from 184 breast cancer patients

GeneName	Methylation level (mean±SD)		Difference^a^	*P* value^b^	Corrected *P* value^c^
	Tumor	Normal			
***ATM***	0.53±0.42	0.55±0.47	-0.02	0.844	1
***ATRX***	28.84±9.49	30.55±6.24	-1.71	**0.036**	0.792
***BLM***	0.63±0.73	0.58±0.38	0.05	0.396	1
***CBX3***	0.52±0.40	0.53±0.84	-0.01	0.906	1
***CMYC***	0.60±0.85	0.57±0.58	0.03	0.787	1
***DAXX***	0.47±0.45	0.45±0.40	0.02	0.521	1
***DKC1***	34.81±21.68	34.18±16.23	0.63	0.726	1
***GAR1***	1.73±1.39	1.77±1.20	-0.04	0.761	1
***HMBOX***	0.60±0.84	0.48±0.60	0.12	0.115	1
***MEN1***	0.55±0.67	0.67±0.78	-0.12	0.133	1
***NBS1***	11.74±5.27	11.53±3.84	0.21	0.672	1
***NHP2***	0.87±0.87	0.68±0.58	0.19	**0.009**	0.216
***NME1***	0.53±0.40	0.47±0.28	0.06	0.129	1
***NOP10***	0.41±0.42	0.45±0.45	-0.04	0.421	1
***OBFC***	0.45±0.60	0.42±0.48	0.03	0.619	1
***PARP1***	0.68±0.66	0.68±0.35	0	0.936	1
***POT1***	0.49±0.52	0.57±0.57	-0.08	0.120	1
***RAD50***	22.52±10.02	16.95±7.02	5.57	**6.33E-08**	**1.77E-06**
***RAD51D***	45.36±9.79	42.81±7.90	2.55	**0.007**	0.175
***RAP1***	0.54±0.37	0.47±0.33	0.07	**0.048**	1
***RECQL5***	0.71±1.12	0.57±0.58	0.14	0.147	1
***RTEL***	66.16±13.66	46.29±10.33	19.87	**3.12E-37**	**9.05E-36**
***TCAB1***	0.45±0.33	0.46±0.28	-0.01	0.883	1
***TEP***	0.91±1.05	0.86±0.77	0.05	0.574	1
***TERC***	1.22±2.33	0.48±0.39	0.74	**3.91E-05**	**1.02E-03**
***TNKS1***	0.52±0.75	0.48±0.66	0.04	0.555	1
***TP53***	0.67±0.66	0.53±0.38	0.14	**0.009**	0.216
***TPP1***	0.63±0.73	0.57±0.39	0.06	0.292	1
***TRF1***	12.03±6.24	9.65±4.37	2.38	**2.98E-05**	**8.05E-04**
29 Genes	8.20±1.28	7.13±0.96	1.07	**4.30E-21**	

SD: Standard deviation; *P* value < 0.05 in bold.

^a^ Difference: the mean of methylation difference between paired tumor and normal tissues

^b^ *P* value calculated by paired t test

^c^ Corrected *P* value using Holm's correction procedure

### Identification of subtype-specific methylation change and its association with clinical characteristics

**Figure 2:**
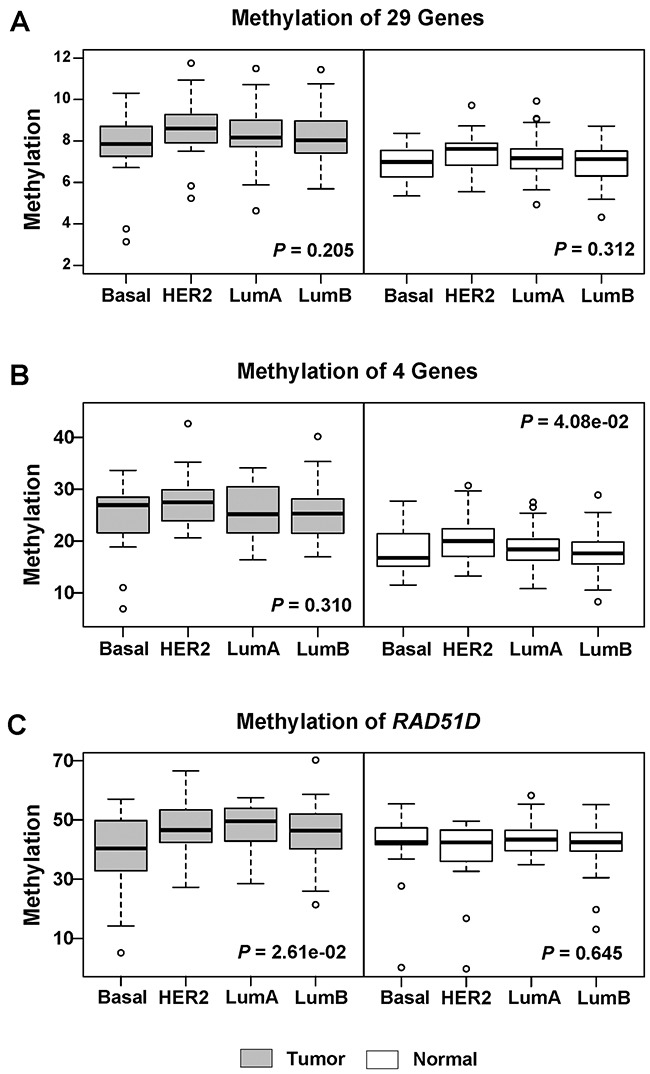
Boxplots stratified by subtypes for methylation levels of candidate genes in 184 tumor and matched normal tissues **(A)** The methylation level was shown for all 29 genes in tumor and normal tissues. **(B)** The methylation level was shown for 4 hyper-methylated genes in tumor and normal tissues. **(C)** The methylation level was shown for *RAD51D* in tumor and normal tissues. *P* value was calculated using Kruskal-Wallis rank sum test. Here, tumor (dark color) and normal (light color) were displayed in different colors.

In four breast cancer subtypes, basal-like patients showed the lowest average methylation level, while HER2-enriched patients showed the highest average methylation level of the 29 genes (Figure 2). Neither the average methylation level of the 29 genes (*P* = 0.205) nor that of the 4 hyper-methylated genes (*P* = 0.310) was significantly different among the 4 breast cancer subtypes. In further analysis of the individual 29 genes methylation in subtypes using the Kruskal–Wallis Rank Sum test, only *RAD51D* gene showed significant methylation difference (*P* = 0.026) among the four subtypes in breast cancer (Figure 2) with the lowest methylation level in basal-like tumor.

Besides, we analyzed the average methylation level of the 29 genes and the 4 hyper-methylated genes in patients with different clinical characteristics. No significant association was found between the average methylation of the 29 genes/4 hyper-methylated genes and clinical characteristics. Then the individual methylation level of the 29 genes was analyzed for the association with patient clinical characteristics. There were several genes showing association of methylation with some clinical characteristics (*P* < 0.05) (Supplementary Table 3-5). However, only the methylation of *TERC* associated with ER status remained significant after Holm's correction (*P* = 0.0203) (Supplementary Table 3).

### Gene expression and their correlation with methylation

In the 29 candidate genes, 4 of them showed significant difference of methylation between the tumor and normal tissues after Holm's correction. To further analyze the potential regulation mechanism, we explored the expression of the 4 hyper-methylated genes in 113 breast cancer patients with enough paired tumor and normal tissues available (Table 2 and Supplementary Figure 1). In paired t test analysis, all of the 4 genes (*RAD50, RTEL, TERC* and *TRF1*) showed significant expression (dCt) difference between tumor and normal tissues (with corrected *P* values of 1.22E-16, 7.78E-05, 1.08E-11 and 5.05E-13 respectively). The 4 genes showed significant hyper-methylation in tumor tissues of the 113 breast cancer patients (Table 2) same as in the 184 samples. It was obvious that all of the 4 genes showed lower expression in tumor compared with that in normal tissues.

**Table 2:** The expression and methylation level of 4 genes in 113 patients and the TCGA database

Gene	Methylation (Mean±SD)			Expression (dCt: Mean±SD)^a^			Methylation (mean) fromMethHC database			Expression (mean RPKM) from MethHC database		
	Tumor	Normal	Corrected*P* value^b^	Tumor	Normal	Corrected*P* value^b^	Tumor	Normal	*P* value^c^	Tumor	Normal	*P* value^c^
***RAD50***	23.73±9.57	16.23±6.04	**9.03E-09**	5.23±1.05	4.15±0.79	**1.22E-16**	7.10	7.21	0.705	1851.4	1678.5	**2.01E-02**
***RTEL***	66.20±14.00	46.08±10.57	**1.06E-21**	5.93±1.29	5.31±1.07	**7.78E-05**	28.77	24.20	**9.53E-12**	343.8	297.0	**8.28E-03**
***TERC***	1.26±2.11	0.48±0.40	**5.65E-04**	6.60±1.62	5.35±1.30	**1.08E-11**	21.27	16.22	**2.42E-06**	0.5	0.3	0.201
***TRF1***	11.97±6.19	9.95±4.46	**5.49E-03**	6.36±1.05	5.37±1.01	**5.05E-13**	4.02	4.34	**2.55E-02**	715.5	597.4	**1.40E-05**

SD: standard deviation; *P* value < 0.05 in bold.

^a^ Higher dCt means lower expression

^b^ Corrected *P* value calculated by paired t test and Holm's correction procedure

^c^ *P* value extracted from MethHC database (uncorrected)

We validated the results with the TCGA data from the MethHC database. *RTEL, TERC* and *TRF1* showed significantly different methylation between tumor and normal tissues with hyper-methylation of *RTEL, TERC* in tumor in TCGA data set (Table 2). *RAD50, RTEL*, and *TRF1* also showed significant difference of expression between tumor and normal tissues in TCGA data but with higher expression in tissues.

We evaluated the methylation level of each gene against their expression level using Spearman's rank correlation test. *RAD50* (*P* = 6.87E-03, *R* = -0.184), *RTEL* (*P* = 3.40E-03, *R* = -0.199) and *TRF1* (*P* = 0.012, *R* = -0.171) showed significant and negative *cis* correlation between promoter methylation and gene expression. While no significant *cis* correlation between the promoter methylation and expression was found for *TERC*.

There is evidence that RTEL gene interacts with TRF1 in protecting telomere ends during replication [36]. Spearman's rank correlation test was used to analyze the correlation of methylation and expression status between these two genes in the 113 patients. Our results indicated that these two genes showed strongly significant correlation of expression status (*P* = 5.42E-17, *R* = 0.523) and significant correlation of methylation status (*P* = 0.011, *R* = 0.171) in breast cancer, both of which were hyper-methylated and down-regulated in expression.

### Evaluation of 4-gene panel as potential diagnostic biomarker for breast cancer prediction

**Figure 3:**
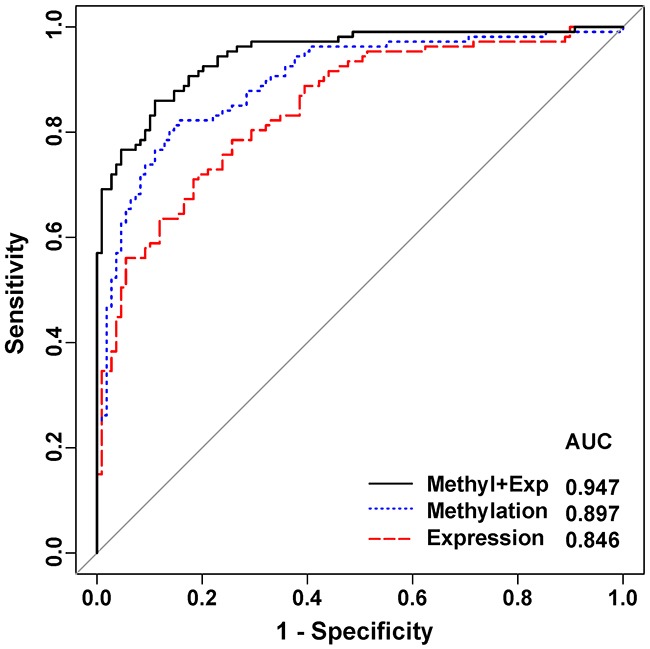
Receiver operating characteristic (ROC) curve analysis in breast cancer detection The curve was obtained by calculating the sensitivity and specificity of the test at every possible cutoff point, and plotting the sensitivity against 1-specificity. The curves were marked for methylation in blue, expression in red, and integrated methylation/expression in black.

The multivariate logistics regression analysis was applied to evaluate the performance of the panel of 4 genes (*RAD50, RTEL, TERC* and *TRF1*) as biomarkers for breast cancer prediction (Table 3). The results showed a good performance with a high level of efficiency for breast cancer prediction using different models of these 4 genes. The ROC analysis (Figure 3) showed that methylation (AUC = 0.897) and expression level (AUC = 0.846) of the 4-gene panel had excellent predictive performance and were able to discriminate tumor from normal tissues. And the integration analysis of methylation and expression using the 4-gene panel showed even better performance in breast cancer detection (Sensitivity = 0.832, Specificity = 0.890, Accuracy = 0.861 and AUC = 0.947). The adjusted estimate of LOOCV prediction error was 0.11 for the integration model (Table 3).

**Table 3:** The predictive performance of logistics regression models using the 4-gene panel in breast tumor classification

Model	CV error ^a^	Sensitivity	Specificity	Accuracy	AUC
**Methylation + Expression**	0.110	0.832	0.890	0.861	0.947
**Methylation**	0.137	0.794	0.862	0.829	0.897
**Expression**	0.170	0.738	0.761	0.750	0.846

^a^ The adjusted estimate of LOOCV prediction error

## DISCUSSION

Dysregulation of telomere and telomerase was a fundamental step in tumorgenesis of almost all kinds of cancers [9]. Promoter methylation is an early event in tumorgenesis and has been considered as a promising biomarker for early detection of cancer [37, 38]. The aberrant promoter methylation of telomere related gene would be a crucial event in tumorgenesis. So far, the promoter methylation of most of telomere related genes has not been studied in breast cancer. DNA repair and telomere maintenance are two crucial processes that protect the genome against instability. The fact that many DNA repair proteins interact with telomeres indicates an important interplay between telomere maintenance and DNA repair [39]. Our study has included some DNA repair genes (Supplementary Table 1). In addition to the 29 genes we studied here, 6 more telomere related genes (*CTC1, MRE11, TRF2, UPF, TIN2*, and *TNKS2*) were beyond the online software's parameters for promoter methylation primer design and were excluded from the study. The other two crucial genes *TERT* and *WRN* have been studied in our previous methylation and expression analysis [40, 41]. To our knowledge, this is the first comprehensive study of methylation on a large number (29) of telomere related genes in a relative large number of samples (184 breast cancer patients).

In the present study, the average methylation level of the 29 telomere related genes was higher in breast tumor than in normal tissue. The 4 genes (*RAD50, RTEL, TERC* and *TRF1*) showed significant hyper-methylation and lower expression in breast cancer. They have different functions in telomere length maintenance, telomerase activity and DNA repair. *RAD50* is a crucial protein enrolled in DNA repair [42]. *RTEL* is a DNA helicase which functions in the stability, protection and elongation of telomeres [43]. *TERC* is a critical component of telomerase complex, which provides RNA template for the telomere elongation [44]. *TRF1* is a component of shelterin complex and functions as an inhibitor of telomerase [45]. Hyper-methylation of these 4 genes may affect their regular functions and results in tumorgenesis.

Aberrant promoter methylation has been indicated to regulate downstream gene expression [46–50]. However lately, a more complex pattern has been reported that gene expression and methylation may be both positively and negatively correlated [41, 51]. Clinically relevant aberrant methylation may serve as a potential biomarker which is not always linked with changes in gene expression [52]. In the analysis of the 4 significantly hyper-methylated genes (*RAD50, RTEL, TERC* and *TRF1*), all of their expression levels were lower in tumor than the matched normal tissues, which suggested a negative correlation trend between methylation and expression. However, in *cis* correlation analysis, only *RAD50, RTEL* and *TRF1* showed significant negative correlation (*P* < 0.05) between methylation and gene expression in our cohort. Although not all of the 4 hyper-methylated genes showed negative *cis* correlation, with the integration of methylation and expression coefficient, the 4-gene panel showed better prediction performance of tumor/normal status than using only methylation or gene expression as parameters. It implied that both methylation and gene expression are crucial in breast cancer tumorgenesis beyond the regulation of methylation on expression.

In the methylation analysis against subtype and clinical characteristics, *RAD51D* showed breast cancer subtype specific methylation pattern and associated with *Ki67* expression level. Besides, three genes (*NME1*, *TERC* and *POT1*) associated with ER status, *NME1* and *RTEL* associated with HER2 status, *ATRX* and *NBS1* associated with the lymph node metastasis, *ATRX* associated with *TP53* mutation status, *TNKS1* associated with the PR status were found in our cohort. It indicated that different methylation patterns of telomere related genes may contribute to the heterogeneity of breast cancer [53].

Our findings validated some of the previously reported biomarkers and provided novel biomarkers for breast cancer detection. In our candidate genes, *RAD50* has been reported to be hyper-methylated in breast cancer patients [28]. Although previous studies indicated some hyper-methylation of *TP53* promoter, the hyper-methylation level was not significant in breast cancer tissues in comparison with adjacent normal tissues [27]. *POT1* was hyper-methylated and can be reactived by 5-aza-2′-deoxycytidine in breast cancer cell line [25]. In our study, *POT1* showed low level of methylation in breast cancer tissues. Hyper-methylation of *RAD50* and *TP53* (*P* = 6.33E-08 and *P* = 0.009 before correction, respectively) in breast cancer was validated in our cohort. However, only *RAD50* remained significant after Holm's correction. We also validated our results with the TCGA data from MethHC database. Three of the 4 hyper-methylated genes (*RTEL, TERC* and *TRF1*) showed significant methylation change in TCGA set (Table 2) except *RAD50*. *RTEL* and *TERC* were also hyper-methylated in tumor in the TCGA data set. These 4 genes showed significant reduced expression in tumor in our cohort. In TCGA data set, *RAD50, RTEL*, and *TRF1* also showed significant gene expression change. However, the direction of up or down regulation of the gene expression is not consistent between these 2 datasets. We noticed that DNA methylation was obtained from Illumina Infinium Human Methylation450 Beadchip and gene expression was obtained from RNASeq in TCGA Data Portal for breast cancer. The discrepancy observed here is most likely related to the differences in detection methods, stage or type of breast tumor, and even the differences in race or ethnicity [41].

The further purpose of our study was to find new biomarkers for breast cancer detection in the telomere related genes. The new biomarker of the 4-gene panel showed good performance in cancer prediction with high sensitivity, specificity accuracy. The model of integrating methylation and expression showed a better performance in breast cancer classification than that using methylation or expression as single parameter. The breast cancer specific methylation and expression pattern, AUC value and their critical role in telomere related functions support these 4 genes as potential biomarkers for breast cancer detection. Since this study is the first comprehensive research on methylation of telomere related genes in breast cancer, cross-validation studies remain to be done for confirmation and further clinical applications of these genes as biomarkers.

It has been reported that RTEL interacts with TRF1 in protecting telomere ends during replication [36]. Our results also showed strongly significant co-expression (*P* = 5.42E-17, *R* = 0.523) and significant co-methylation (*P* = 0.011, *R* = 0.171) of these two genes using Spearman's rank correlation coefficient test. RTEL also functions in maintenance of general genome integrity [54]. It may be through some of the non telomere related mechanisms that it exerts an influence on tumorgenesis. The interaction of these two genes in breast cancer is worth further investigation.

In summary, the present study provided a comprehensive evaluation of methylation pattern of 29 telomere related genes in breast cancer. Consequent confirmation of our results could lead to a better understanding of epigenetic characteristic of the telomere related genes and promoting the clinical application of these methylation biomarkers for early detection and treatment monitoring of breast cancer.

## MATERIALS AND METHODS

### Patients and tumor specimens

Fresh frozen primary breast tumor and matched adjacent normal tissues (located at least 2 cm away from the site of tumor tissue) were obtained from 184 patients with no prior chemotherapy or radiotherapy who underwent surgical resection of the breast tumors at Xiangya Hospital, Central South University from 2013 to 2015. All breast specimens were reviewed by experienced pathologists. The clinicopathological characteristics of 184 patients were summarized in Table 4. The tumors were classified based on the guideline of St Gallen International Expert Consensus [55]. The study was approved by the Ethics Committee of Central South University, Changsha, China. All participants provided written informed consent for participation in the study.

**Table 4:** Clinicopathological characteristics of 184 breast cancer patients

Characteristics	Subtypes	Number of Patients, n (%)
**Molecular subtype**	Basal-like	22 (12.0)
	HER2-enriched	21 (11.4)
	Luminal A	46 (25.0)
	Luminal B	83 (45.1)
	Unknown	12 (6.5)
**ER status**	Positive (+)	134 (72.8)
	Negative (-)	50 (27.2)
**PR status**	Positive (+)	111 (60.3)
	Negative (-)	73 (39.7)
**HER2 status**	Positive (+)	98 (53.3)
	Negative (-)	58 (31.5)
	Unknown	28 (15.2)
**Lymph node metastasis**	Yes	69 (37.5)
	No	115 (62.5)
**Age**	≥50	99 (53.8)
	[35-50)	80 (43.4)
	<35	5 (2.7)
***Ki67* level**	<10%	46 (25.0)
	10%-25%	71 (38.6)
	>25%	67 (36.4)
***TP53* mutation**	Positive (+)	138 (75.0)
	Negative (-)	46 (25.0)

### DNA bisulfite conversion and RNA reverse transcription

DNA and total RNA was extracted from fresh frozen tissue samples as previously described [40, 41]. Sodium bisulfite conversion of 500 ng genomic DNA was carried out using the EZ DNA Methylation-Lightning^TM^ Kit (Zymo Research, Irvine, CA, USA) according to the manufacturer's instruction. For cDNA synthesis, 500 ng total RNA was reverse transcribed using a RevertAid 1st Strand cDNA synthesis kit (Thermo Scientific, CA, USA) according to the manufacturer's instruction.

### Candidate gene selection and primer design

Firstly we selected the genes (*DKC1, POT1, RAP1, TERC, TERT, TIN2, TPP1, TRF1, TRF2*) encoding telomerase and core shelterin complex from the telomerase database (http://telomerase.asu.edu/reviews.html) and recently published papers [8, 56]. Then we selected the genes encoding core factors, which interact with the telomerase and shelterin complex composition (*ATM, ATRX, ATRX, BLM, CBX3, CMYC, CTC1, DAXX, GAR1, HMBOX1, MEN1, MRE11, NBS1, NHP2, NME1, OBFC, PARP1, RAD50, RAD51D, RECQL5, RTEL, TCAB1, TEP, TNKS1, TNKS2, TP53, UPF, WRN*) based on published papers [56–60]. These genes involved in telomere maintenance, as well as genes that are transiently associated with the telomere, and genes involved in DNA repair and helicase genes [56–60]. *TERT* and *WRN* have been studied in our previous methylation/expression studies [40, 41] and excluded from analysis here. The 5′ promoter sequence of candidate genes was obtained from UCSC genome browser (http://genome.ucsc.edu/cgi-bin/hgGateway). Target-specific bisulfite sequencing PCR primers (BSP) were designed using the online software, Methprimer (http://www.urogene.org/methprimer/). However, the promoter sequences of *CTC1*, *MRE11*, *TIN2*, *TNKS2*, *TRF2*, and *UPF* were beyond the online software's parameters for methylation primer design and were excluded from the study. Finally, 29 telomere related genes were selected for further investigation in the present study. Functional categories of the 29 candidate genes were analyzed using the DAVID functional annotation tool (http://david.abcc.ncifcrf.gov) and summarized in Supplementary Table 1. After methylation primer design, universal sequencing tags were added to the 5′-end of the forward and reverse primer sequences by following the User Guide of Access Array^TM^ System for Illumina Sequencing Systems (Fluidigm, South San Franciso, CA, USA). For expression analysis, cDNA sequence was obtained from the Consensus CDS (CCDS) (https://www.ncbi.nlm.nih.gov/CCDS/CcdsBrowse.cgi). Gene expression primers of candidate genes and reference gene *GAPDH* were designed using Primer 3. All primers were validated by PCR and products were confirmed on agarose gels. The amplification efficiency of the gene expression primers were between 90% and 110%. The primer sequences were displayed in Supplementary Table 2.

### High-throughput microfluidic PCR for target sequence amplification and next generation sequencing

As our previous studies, the 48.48 Access Array^TM^ system (Fluidigm, South San Franciso, CA, USA) and Access Array Barcode Library was used for target enrichment and sequencing libraries construction [40, 41]. The product size distribution was examined by Agilent Bioanalyzer 2100. The purified libraries were quantified with Qubit® dsDNA HS Assay Kit (Life Technologies, CA, USA) and sequenced on a MiSeq sequencer using MiSeq Reagent Kit v2 (500 cycles). The methylation status and methylation level of each analyzed CpG-site were returned from trimmed read data. The methylation level for each gene was assigned by averaging the methylation level of all CpG sites in the promoter amplicon for each sample as previously reported [40].

### Expression analysis

To explore the expression variation of the hyper-methylated genes, real-time fluorescence quantitative polymerase chain reaction (qPCR) was performed on a CFX96™ Real-Time PCR Detection System (Bio-Rad, CA, USA). In the 184 patient specimens, total 113 patients were available for expression analysis due to the limitation of tissue block. All the qPCR experiments were performed in triplicates. The qPCR mixture consisted of 2 μL of cDNA sample, 2 μL nuclease-free water, 5 μL 2 × SYBR Green PCR master mix (Roche, IN, USA), and 1 μL of each gene specific primer (2 μm). The PCR cycling conditions were: 1 cycle of 95 °C per 10 min, 40 cycles of 95 °C per 5 s, 60°C per 30 s, 72 °C per 30 s, followed by dissociation curve analysis (65–95 °C: increment 0.5 °C for 5 s) to verify the amplification of a single product. The threshold cycle (Ct) value was determined using the auto setting on the CFX Sequence Detection System. The gene expression difference between tumor and normal tissues was determined using delta Ct (dCt) as: Ct (Target gene) - Ct (*GAPDH*), where a larger dCt value means lower expression level.

### Statistical analysis

The paired t test was used to determine the difference in methylation and expression level between tumor and normal tissues. Spearman's rank correlation coefficient test was applied to analyze association between methylation and expression in tumor and normal tissues. The Kruskal–Wallis rank sum test was performed to examine the correlations between gene methylation and expression levels against clinicopathological characteristics and subtypes. It was considered statistically significant if the *P* value was less than 0.05 and adjusted using the Holm's correction procedure [23]. A multivariate logistics regression analysis was applied to classify tumor/normal status. The predictive performance using the logistics regression models for the selected panel was evaluated based upon sensitivity, specificity, and accuracy measurements. And the prediction error using leave one out cross validation (LOOCV) method was also estimated as a performance measurement for these models. AUCs and receiver operating characteristic (ROC) analysis were also performed. All statistical analysis was done in R environment (version 3.1.0).

### Validation in the cancer genome atlas (TCGA) dataset

To verify if the methylation and expression patterns of the 4 hyper-methylated telomere related genes were characteristics of breast cancer, we downloaded the methylation and gene expression data of breast cancer for the 4 genes from MethHC website (http://MethHC.mbc.nctu.edu.tw/). MethHC is a newly developed database comprising a systematic integration of a large collection of gene methylation and expression data of human cancers from The Cancer Genome Atlas (TCGA) [56]. We compared our results with the corresponding TCGA data of breast cancer to validate our findings.

## SUPPLEMENTARY MATERIALS FIGURES AND TABLES













## Abbreviations

ALT: Alternative lengthening of telomeres
AUC: Area Under the ROC Curve
CCDS: Consensus coding sequence
ER: Estrogen receptor
HER2: Human epidermal growth factor receptor 2
LOOCV: Leave one out cross validation
PR: Progesterone receptor
qPCR: Quantitative polymerase chain reaction
ROC: Receiver operating characteristic
TCGA: The Cancer Genome Atlas
TERT: Telomerase reverse transcriptase
