# Supplementary material for: Integrated analysis of promoter methylation and expression of telomere related genes in breast cancer
Source: Oncotarget. 2017 Mar 9;8(15):25442–54. doi: 10.18632/oncotarget.16036 (PMC5421942; doi:10.18632/oncotarget.16036)
Supplement: Supplementary file 1 [file oncotarget-08-25442-s001.pdf]

Integrated analysis of promoter methylation and expression of telomere related genes in breast cancer

SUPPLEMENTARY MATERIALS

SUPPLEMENTARY FIGURE AND TABLES

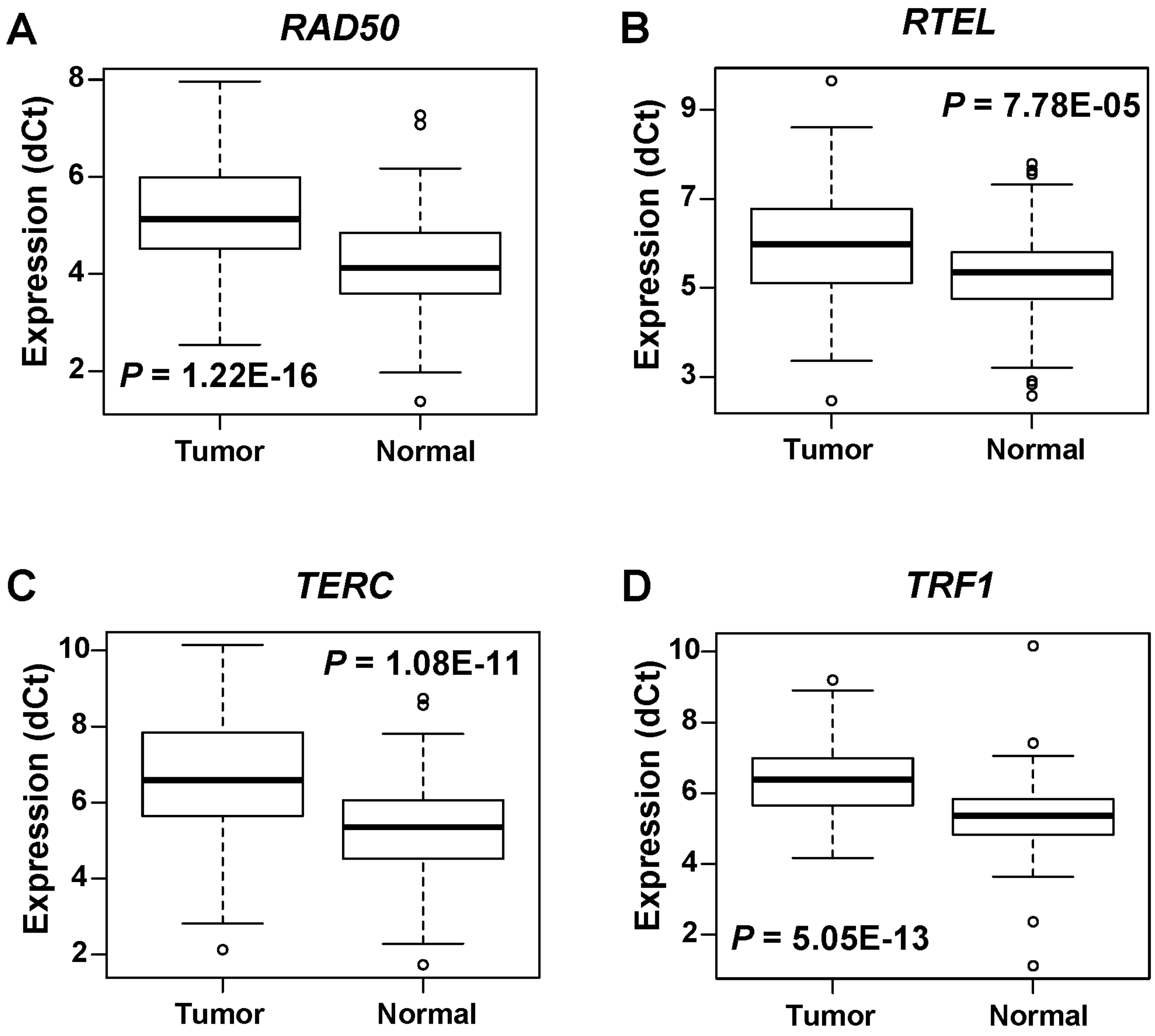

Supplementary Figure 1: Boxplots for expression levels of four hyper-methylated genes in 113 tumor and matched normal tissues.

Supplementary Table 1: David functional annotation of candidate genes.

See Supplementary File 1

Supplementary Table 2: Sequences of the bisulfite sequencing and gene expression primers*.

See Supplementary File 2

Supplementary Table 3: Correlation of the tumor methylation level of the 29 genes with ER/PR/HER2 status in breast cancer patients.

See Supplementary File 3

Supplementary Table 4: Correlation of the tumor methylation level of the 29 genes with *P53* mutation and lymph node metastasis status in breast cancer patients.

See Supplementary File 4

Supplementary Table 5: Correlation of the tumor methylation level with Ki67 expression level and age in breast cancer patients.

See Supplementary File 5
