# Supplementary material for: Integrated analysis of promoter methylation and expression of telomere related genes in breast cancer
Source: Oncotarget. 2017 Mar 9;8(15):25442–54. doi: 10.18632/oncotarget.16036 (PMC5421942; doi:10.18632/oncotarget.16036)
Supplement: Supplementary file 2 [file oncotarget-08-25442-s002.docx]

**Supplementary Table 1. David functional annotation of candidate genes**

| Gene | nuclear chromosome, telomeric region | chromosome, telomeric region | telomerase holoenzyme complex | telomere maintenance via telomerase | telomere maintenance | chromosome, centromeric region | h_telPathway:Telomeres, Telomerase, Cellular Aging, and Immortality | DNA repair |
| --- | --- | --- | --- | --- | --- | --- | --- | --- |
| *ATM1* | x | x |  | x | x |  |  | x |
| *ATRX* | x |  |  |  |  |  |  | x |
| *BLM* |  | x |  |  |  |  |  | x |
| *CBX3* | x |  |  |  |  | x |  |  |
| *CMYC* |  |  |  |  |  |  | x |  |
| *DAXX* |  |  |  |  |  | x |  |  |
| *DKC1* |  |  | x | x |  |  |  |  |
| *GAR1* | x |  | x | x |  |  |  |  |
| *HMBOX1* | x |  | x |  |  |  |  |  |
| *MEN1* | x |  |  |  |  |  |  | x |
| *NBS1* | x | x |  |  | x |  |  | x |
| *NHP2* | x |  | x | x |  |  |  |  |
| *NME1* |  |  |  |  |  |  |  |  |
| *NOP10* |  |  | x | x |  |  |  |  |
| *OBFC1* | x |  |  |  | x |  |  |  |
| *PARP1* | x |  |  |  |  |  |  | x |
| *POT1* | x | x |  | x | x |  |  |  |
| *RAD50* | x |  |  | x | x |  |  | x |
| *RAD51D* | x | x |  |  | x |  |  | x |
| *RAP1* | x | x |  | x | x |  |  |  |
| *RECQL5* |  |  |  |  |  |  |  | x |
| *RTEL* |  |  |  |  | x |  |  | x |
| *TCAB1* |  |  | x | x |  |  |  |  |
| *TEP* |  | x | x |  |  |  | x |  |
| *TERC* |  |  |  |  |  |  |  |  |
| *TNKS1* | x | x |  |  |  | x | x |  |
| *TP53* |  |  |  |  |  |  | x |  |
| *TPP1* | x |  |  |  | x |  |  |  |
| *TRF1* | x | x |  | x | x |  | x |  |
| Total | 17 | 9 | 7 | 10 | 10 | 3 | 5 | 10 |
