# Supplementary material for: Integrated analysis of promoter methylation and expression of telomere related genes in breast cancer
Source: Oncotarget. 2017 Mar 9;8(15):25442–54. doi: 10.18632/oncotarget.16036 (PMC5421942; doi:10.18632/oncotarget.16036)
Supplement: Supplementary file 3 [file oncotarget-08-25442-s003.docx]

**Supplementary Table 2. Sequences of the bisulfite sequencing and gene expression primers***

| **Gene name** | **F-primer (5'-3')** | **R-primer (5'-3')** | **CpG sites in product** | **Application** |
| --- | --- | --- | --- | --- |
| *ATM1* | AGGAATTATAATAAGGAATAAGATTTAGGT | CCATATCCACCAATAACCAAC | 12 | Bisulfite sequencing |
| *ATRX* | AGGTTAATTGGGATTAAATTGGATT | ATTAACTACCTCCTCCAAAACACAA | 5 |  |
| *BLM* | TGGAGGTATTTGAGTGTGTGTTTAT | AACTTACCAATTCCCACAAACC | 8 |  |
| *CBX3* | TGTGGAATTGATTTTTGGTGTATAG | AACTTTTCTACTTAACAACCTCAAC | 9 |  |
| *CMYC* | GTGGGGTATAGATTGGTAGAGAGTAG | CACAACTATCTAAATTAAATACCTTCCAC | 10 |  |
| *DAXX* | TTTGAAATTTTGTATAGTTTTGGAAAGT | CAACCAATAAAACTCTTTTCACC | 10 |  |
| *DKC1* | TGGATGGGAATTGTAAGTAGTAAAG | CAACCTAATTAACCCAAACTAAAAAC | 16 |  |
| *GAR1* | TGAGATTATAGGATAAAAGAAGAGGTATAG | AACACTCACTAATTTATTACTAACTTCAAA | 10 |  |
| *HMBOX1* | GGTTTGGGGTTTATGGTGTTT | CCTTATCTCAATCACATCAAACTAACTAT | 13 |  |
| *MEN1* | TTTTGGTAGTGTTTAAAAGTATTTGTTTT | AAATTAACCCTTCCCTTCAAAC | 11 |  |
| *NBS1* | TGGTTGGAAAAGGAATAGTATAATTTG | CCATTACTAAATTCCAAAAAAATACCTAC | 4 |  |
| *NHP2* | GAGGGAATGTGAGGTAAGGG | CCACTATACCCCAACCTAAACAATA | 14 |  |
| *NME1* | TTGATAGGTTAGAAAAGGTGAATAAATATA | AAAACAACTTTCTACAATTTAACTTCC | 13 |  |
| *NOP10* | GTTTTAGTTTGTAAAGTTGTAAGTAGGG | AAAATCTCTCTCTCCTAACACTCC | 7 |  |
| *OBFC* | TTAAATATTAATTTTTATTTTGGGAAGATA | CCAAATCTCAAAACTCCTAAAACTAC | 12 |  |
| *PARP1* | TTTGATAGATTGTTGATGTTTGGT | AAACTACTAACTCAACCCAAACCC | 24 |  |
| *POT1* | GAGAATTGGATATTAAAAGAAAGGTTTT | CAAAACCAAAATACAAACCCC | 12 |  |
| *RAD50* | TTATATGTTATGTGATTATTGGAAATTAT | TTCCCAAAACTTAATCCTAAAACTC | 8 |  |
| *RAD51D* | ATTGTGAGTAAGGGAAAGATAGATAATG | AACTAAAATCTCACCAAAACACTCC | 7 |  |
| *RAP1* | TAGTTAGTTGAGTTTTGTGTGTTAGG | AAATCTTTACCCAAATCCATC | 14 |  |
| *RECQL5* | AGTTTAGGGGAAATAGGTAGTGGTT | CAAAATAATCATTATACTCCAAACACC | 9 |  |
| *RTEL* | GGGTTGGGTGTTAGTGAGTGT | CAACTCCCATACCCCAAAAA | 12 |  |
| *TCAB1* | AGGAGTTTTAGGGTTTGATGG | ACTTCCCTCCTCTTCTAAAAATAAAC | 12 |  |
| *TEP* | TTTGATTGATTGATGTTTATATTTGGT | ATTTTCTCCTATCCCTAAAACTTCC | 8 |  |
| *TERC* | AAAATTTGTAGAGTAGGAATTAAGTTG | CCCCAAACCTAACTAACTAAAC | 7 |  |
| *TNKS1* | AGGAAATGTTTTTTATTTAGGTAGAAGTT | AACTCCCCTAACACTATCACTAC | 8 |  |
| *TP53* | GAGGTTTTTGGTATAAAGTTGGATAGT | CTCTAACTTACAAAATTTTCCACCC | 7 |  |
| *TPP1* | TGGGTTTATTTAGTGTTTGATTGGT | CACCAAACTTAAAATCCCATTCTAC | 6 |  |
| *TRF1* | TAGTAGAATAGGAATTTTGGGAGT | AATACAACCTTAACTAAAAC | 6 |  |
| *RAD50* | TGGATATGCGAGGACGATG | TGTTGGCTCATCCAAGGCA |  | Gene expression analysis |
| *RTEL* | CATCGATGCTGTTGAGCTGC | GGATGATCTGGTCCAGCGAG |  |  |
| *TERC* | CATGTGTGAGCCGAGTCCTG | GAAGAGGAACGGAGCGAGTC |  |  |
| *TRF1* | GTCTGCGGTAACTGAATCCTCA | TTGTTGCTGGGTTCCATGTT |  |  |
| *GAPDH* | CCTCTCCCCAGCCAAAGAAG | TGACCCTTTTTGGACTTCAG |  |  |

* For the bisulfite sequencing primers, universal sequencing tags were added to the 5’-end of the forward and reverse primers by following the User Guide of Access Array^TM^ System for Illumina Sequencing Systems (Fluidigm, South San Franciso, CA, USA).
