# Supplementary material for: Integrated analysis of promoter methylation and expression of telomere related genes in breast cancer
Source: Oncotarget. 2017 Mar 9;8(15):25442–54. doi: 10.18632/oncotarget.16036 (PMC5421942; doi:10.18632/oncotarget.16036)
Supplement: Supplementary file 4 [file oncotarget-08-25442-s004.docx]

**Supplementary Table 3. Correlation of the tumor methylation level of the 29 genes with ER/PR/HER2 status in breast cancer patients**

| **Gene** | **ER Positive (n=134)** | **ER Negative (n=50)** | ***P* value^1^** | **Corrected *P* value^2^** | **PR Positive (n=111)** | **PR Negative (n=73)** | ***P* value^1^** | **Corrected *P* value^2^** | **HER2 Positive (n=58)** | **HER2 Negative (n=98)** | ***P* value^1^** | **Corrected *P* value^2^** |
| --- | --- | --- | --- | --- | --- | --- | --- | --- | --- | --- | --- | --- |
| *ATM1* | 0.53±0.45 | 0.45±0.25 | 0.1852 | 1 | 0.55±0.47 | 0.44±0.29 | 0.0899 | 1 | 0.52±0.32 | 0.52±0.48 | 0.8899 | 1 |
| *ATRX* | 28.08±9.7 | 30.67±8.75 | 0.0891 | 1 | 27.83±9.49 | 30.27±9.44 | 0.0907 | 1 | 30.73±9.35 | 28±8.96 | 0.0758 | 1 |
| *BLM* | 0.64±0.82 | 0.58±0.43 | 0.5242 | 1 | 0.58±0.45 | 0.7±1.03 | 0.3791 | 1 | 0.63±0.52 | 0.58±0.49 | 0.5612 | 1 |
| *CBX3* | 0.49±0.36 | 0.6±0.5 | 0.1642 | 1 | 0.47±0.36 | 0.59±0.45 | 0.0649 | 1 | 0.51±0.46 | 0.53±0.39 | 0.7949 | 1 |
| *CMYC* | 0.57±0.76 | 0.64±1.05 | 0.6624 | 1 | 0.61±0.85 | 0.55±0.86 | 0.6431 | 1 | 0.62±1.05 | 0.57±0.8 | 0.7565 | 1 |
| *DAXX* | 0.46±0.36 | 0.52±0.63 | 0.5312 | 1 | 0.47±0.36 | 0.49±0.57 | 0.7477 | 1 | 0.47±0.59 | 0.47±0.39 | 0.936 | 1 |
| *DKC1* | 34.74±20.92 | 36.13±22.79 | 0.7104 | 1 | 35.34±21.06 | 35.08±22.01 | 0.9392 | 1 | 33.47±21.56 | 35.71±22.85 | 0.5405 | 1 |
| *GAR1* | 1.69±1.4 | 1.78±1.35 | 0.6917 | 1 | 1.8±1.26 | 1.6±1.56 | 0.3726 | 1 | 1.56±1.12 | 1.63±1.18 | 0.6825 | 1 |
| *HMBOX1* | 0.64±0.94 | 0.5±0.49 | 0.1981 | 1 | 0.68±1.01 | 0.48±0.49 | 0.0794 | 1 | 0.53±0.97 | 0.61±0.8 | 0.6082 | 1 |
| *MEN1* | 0.54±0.58 | 0.58±0.9 | 0.7642 | 1 | 0.51±0.59 | 0.62±0.78 | 0.3314 | 1 | 0.42±0.43 | 0.64±0.81 | **0.0308** | 0.8624 |
| *NBS1* | 11.5±5.5 | 11.86±4.96 | 0.6711 | 1 | 11.67±5.41 | 11.5±5.34 | 0.8386 | 1 | 11.92±5.03 | 11.32±5.63 | 0.4886 | 1 |
| *NHP2* | 0.92±0.91 | 0.74±0.74 | 0.1786 | 1 | 0.94±0.91 | 0.77±0.8 | 0.1859 | 1 | 0.97±1.05 | 0.75±0.62 | 0.1507 | 1 |
| *NME1* | 0.55±0.44 | 0.44±0.25 | **0.0368** | 0.99 | 0.52±0.43 | 0.51±0.35 | 0.8531 | 1 | 0.53±0.4 | 0.48±0.38 | 0.4836 | 1 |
| *NOP10* | 0.41±0.43 | 0.42±0.4 | 0.8192 | 1 | 0.42±0.45 | 0.41±0.38 | 0.8662 | 1 | 0.43±0.42 | 0.41±0.46 | 0.7231 | 1 |
| *OBFC1* | 0.4±0.49 | 0.57±0.82 | 0.2002 | 1 | 0.45±0.64 | 0.45±0.54 | 0.9886 | 1 | 0.32±0.35 | 0.47±0.6 | 0.0532 | 1 |
| *PARP1* | 0.64±0.32 | 0.75±1.17 | 0.5213 | 1 | 0.64±0.32 | 0.72±0.97 | 0.5177 | 1 | 0.66±1.09 | 0.67±0.32 | 0.9588 | 1 |
| *POT1* | 0.52±0.57 | 0.38±0.27 | **0.0275** | 0.77 | 0.5±0.56 | 0.46±0.42 | 0.5782 | 1 | 0.41±0.39 | 0.51±0.55 | 0.2196 | 1 |
| *RAD50* | 22.58±9.91 | 22.16±10.26 | 0.809 | 1 | 21.81±9.73 | 23.48±10.39 | 0.2841 | 1 | 23.34±9.86 | 22.6±10.4 | 0.6589 | 1 |
| *RAD51D* | 46.34±8.61 | 42.51±12.22 | 0.0523 | 1 | 46.44±8.49 | 43.54±11.42 | 0.0716 | 1 | 46.25±9.16 | 44.66±10.81 | 0.3395 | 1 |
| *RAP1* | 0.54±0.36 | 0.53±0.41 | 0.9389 | 1 | 0.55±0.37 | 0.52±0.38 | 0.5593 | 1 | 0.52±0.34 | 0.55±0.4 | 0.5986 | 1 |
| *RECQL5* | 0.7±1.26 | 0.73±0.6 | 0.824 | 1 | 0.76±1.36 | 0.62±0.6 | 0.337 | 1 | 0.65±0.64 | 0.76±1.42 | 0.5137 | 1 |
| *RTEL* | 64.97±13.52 | 68.66±15.87 | 0.1532 | 1 | 65.52±12.79 | 66.92±16.15 | 0.5393 | 1 | 68.82±13.15 | 63.75±15.13 | **0.0297** | 0.8613 |
| *TCAB1* | 0.43±0.27 | 0.51±0.45 | 0.217 | 1 | 0.44±0.28 | 0.46±0.39 | 0.7141 | 1 | 0.39±0.28 | 0.48±0.35 | 0.0755 | 1 |
| *TEP* | 0.87±1.02 | 1.11±1.28 | 0.2517 | 1 | 0.82±0.76 | 1.12±1.45 | 0.1062 | 1 | 1.19±1.55 | 0.81±0.79 | 0.0854 | 1 |
| *TERC* | 1.46±2.66 | 0.59±0.75 | **0.0007** | **0.0203** | 1.39±2.59 | 1±1.91 | 0.241 | 1 | 0.8±1.1 | 1.43±2.84 | 0.0508 | 1 |
| *TNKS1* | 0.54±0.79 | 0.51±0.67 | 0.8001 | 1 | 0.63±0.95 | 0.41±0.28 | **0.029** | 0.841 | 0.42±0.52 | 0.59±0.87 | 0.1531 | 1 |
| *TP53* | 0.63±0.53 | 0.81±0.92 | 0.2022 | 1 | 0.61±0.54 | 0.79±0.81 | 0.085 | 1 | 0.63±0.52 | 0.71±0.78 | 0.441 | 1 |
| *TPP1* | 0.65±0.81 | 0.58±0.52 | 0.5264 | 1 | 0.57±0.49 | 0.73±1 | 0.2037 | 1 | 0.63±0.54 | 0.58±0.45 | 0.507 | 1 |
| *TRF1* | 11.74±6.1 | 12.46±6.45 | 0.5091 | 1 | 11.63±6.22 | 12.39±6.17 | 0.4236 | 1 | 13.49±6.39 | 11.4±6.33 | 0.0525 | 1 |
| 29 Genes | 8.18±1.13 | 8.28±1.63 | 0.7105 | / | 8.2±1.09 | 8.25±1.52 | 0.8048 | / | 8.36±1.21 | 8.08±1.36 | 0.1862 | / |

^1^*P* values calculated with Kruskal-Wallis Rank Sum Test, ^2^ Holm's corrected *P* values, *P*<0.05 in bold
