# Supplementary material for: Integrated analysis of promoter methylation and expression of telomere related genes in breast cancer
Source: Oncotarget. 2017 Mar 9;8(15):25442–54. doi: 10.18632/oncotarget.16036 (PMC5421942; doi:10.18632/oncotarget.16036)
Supplement: Supplementary file 5 [file oncotarget-08-25442-s005.docx]

**Supplementary Table 4. Correlation of the tumor methylation level of the 29 genes with *P53* mutation and lymph node metastasis status in breast cancer patients**

| **Gene** | ***P53* Positive (n=138)** | ***P53* Negative (n=46)** | ***P* value^1^** | **Corrected *P* value^2^** | **Node positive (n=69)** | **Node negative (n=115)** | ***P* value^1^** | **Corrected *P* value^2^** |
| --- | --- | --- | --- | --- | --- | --- | --- | --- |
| *ATM1* | 0.52±0.26 | 0.49±0.44 | 0.5909 | 1 | 0.53±0.47 | 0.5±0.37 | 0.6749 | 1 |
| *ATRX* | 31.86±8.8 | 27.93±9.73 | **0.0201** | 0.5829 | 26.92±10.17 | 30.02±9.02 | **0.0398** | 1 |
| *BLM* | 0.53±0.42 | 0.65±0.81 | 0.2569 | 1 | 0.64±0.58 | 0.62±0.83 | 0.8831 | 1 |
| *CBX3* | 0.59±0.49 | 0.5±0.38 | 0.326 | 1 | 0.53±0.41 | 0.51±0.4 | 0.8619 | 1 |
| *CMYC* | 0.71±1.33 | 0.54±0.69 | 0.4893 | 1 | 0.62±0.96 | 0.58±0.77 | 0.737 | 1 |
| *DAXX* | 0.51±0.66 | 0.46±0.37 | 0.6641 | 1 | 0.55±0.57 | 0.43±0.36 | 0.1456 | 1 |
| *DKC1* | 36.07±19.12 | 34.31±22.44 | 0.6293 | 1 | 36.54±23.04 | 34.35±20.43 | 0.5174 | 1 |
| *GAR1* | 1.95±1.37 | 1.68±1.41 | 0.2997 | 1 | 1.58±1.1 | 1.82±1.54 | 0.2207 | 1 |
| *HMBOX1* | 0.47±0.37 | 0.58±0.75 | 0.1809 | 1 | 0.63±0.74 | 0.58±0.9 | 0.7142 | 1 |
| *MEN1* | 0.41±0.44 | 0.59±0.73 | 0.0556 | 1 | 0.49±0.71 | 0.59±0.65 | 0.3644 | 1 |
| *NBS1* | 12.56±5.36 | 11.47±5.39 | 0.2746 | 1 | 10.03±5.01 | 12.52±5.34 | **0.0019** | 0.0551 |
| *NHP2* | 0.85±0.75 | 0.86±0.9 | 0.9684 | 1 | 0.92±0.94 | 0.83±0.83 | 0.52 | 1 |
| *NME1* | 0.51±0.33 | 0.54±0.43 | 0.6877 | 1 | 0.52±0.4 | 0.53±0.41 | 0.8529 | 1 |
| *NOP10* | 0.36±0.29 | 0.42±0.46 | 0.2881 | 1 | 0.4±0.5 | 0.43±0.38 | 0.705 | 1 |
| *OBFC1* | 0.49±0.71 | 0.44±0.58 | 0.7165 | 1 | 0.4±0.54 | 0.48±0.63 | 0.4187 | 1 |
| *PARP1* | 0.64±0.27 | 0.69±0.74 | 0.5656 | 1 | 0.61±0.31 | 0.71±0.81 | 0.2609 | 1 |
| *POT1* | 0.37±0.32 | 0.52±0.55 | 0.0361 | 1 | 0.42±0.42 | 0.52±0.56 | 0.1735 | 1 |
| *RAD50* | 24.34±7.53 | 21.89±10.53 | 0.1144 | 1 | 21.98±10.44 | 22.77±9.62 | 0.6163 | 1 |
| *RAD51D* | 44.44±10.84 | 45.3±9.43 | 0.6604 | 1 | 45±10.72 | 45.67±9.3 | 0.6726 | 1 |
| *RAP1* | 0.55±0.43 | 0.53±0.36 | 0.7498 | 1 | 0.55±0.39 | 0.53±0.36 | 0.6968 | 1 |
| *RECQL5* | 0.64±0.58 | 0.73±1.25 | 0.5231 | 1 | 0.88±1.67 | 0.59±0.57 | 0.1754 | 1 |
| *RTEL* | 65.41±13.23 | 66.23±14.45 | 0.7404 | 1 | 65.64±15.34 | 65.88±13.48 | 0.915 | 1 |
| *TCAB1* | 0.44±0.37 | 0.45±0.32 | 0.9233 | 1 | 0.48±0.34 | 0.42±0.28 | 0.263 | 1 |
| *TEP* | 0.76±0.82 | 1±1.18 | 0.153 | 1 | 0.87±0.94 | 0.97±1.18 | 0.5149 | 1 |
| *TERC* | 0.77±1.22 | 1.32±2.5 | 0.0625 | 1 | 1.16±2.15 | 1.29±2.46 | 0.7106 | 1 |
| *TNKS1* | 0.75±1.03 | 0.47±0.65 | 0.1201 | 1 | 0.55±0.79 | 0.53±0.74 | 0.9172 | 1 |
| *TP53* | 0.7±0.55 | 0.67±0.7 | 0.7588 | 1 | 0.8±0.87 | 0.6±0.49 | 0.0944 | 1 |
| *TPP1* | 0.6±0.45 | 0.64±0.81 | 0.6907 | 1 | 0.63±0.5 | 0.64±0.86 | 0.9695 | 1 |
| *TRF1* | 13.65±6.11 | 11.62±6.33 | 0.0834 | 1 | 12.89±7 | 11.53±5.77 | 0.1844 | 1 |
| 29 Genes | 8.5±1.26 | 8.11±1.29 | 0.0961 | / | 8.09±1.32 | 8.28±1.26 | 0.3256 | / |

^1^*P* values calculated with Kruskal-Wallis Rank Sum Test, ^2^ Holm's corrected *P* values, *P*<0.05 in bold
