# Supplementary material for: Integrated analysis of promoter methylation and expression of telomere related genes in breast cancer
Source: Oncotarget. 2017 Mar 9;8(15):25442–54. doi: 10.18632/oncotarget.16036 (PMC5421942; doi:10.18632/oncotarget.16036)
Supplement: Supplementary file 6 [file oncotarget-08-25442-s006.docx]

**Supplementary Table 5. Correlation of the tumor methylation level with Ki67 expression level and age in breast cancer patients**

| **Gene** | **Ki67<10% (n=46)** | **Ki67=10%-25% (n=71)** | **Ki67>25% (n=67)** | ***P* value^1^** | **Corrected *P* value^2^** | **Age>50 (n=99)** | **35 =< Age<50 (n=80)** | **Age<35 (n=5)** | ***P* value^1^** | **Corrected *P* value^2^** |
| --- | --- | --- | --- | --- | --- | --- | --- | --- | --- | --- |
| *ATM1* | 0.51±0.33 | 0.58±0.53 | 0.43±0.29 | 0.2222 | 1 | 0.55±0.44 | 0.45±0.44 | 0.47±0.35 | 0.1498 | 1 |
| *ATRX* | 29.29±7.65 | 28.75±10.03 | 28.68±10.28 | 0.7334 | 1 | 28.29±9.11 | 33.21±6.12 | 29.26±10.09 | 0.2935 | 1 |
| *BLM* | 0.61±0.54 | 0.69±1.01 | 0.58±0.49 | 0.5502 | 1 | 0.63±0.85 | 0.35±0.13 | 0.64±0.59 | 0.5178 | 1 |
| *CBX3* | 0.5±0.42 | 0.47±0.29 | 0.58±0.48 | 0.8634 | 1 | 0.53±0.41 | 0.48±0.42 | 0.5±0.39 | 0.5583 | 1 |
| *CMYC* | 0.47±0.41 | 0.75±1.23 | 0.5±0.49 | 0.9587 | 1 | 0.61±0.86 | 0.68±0.76 | 0.57±0.85 | 0.844 | 1 |
| *DAXX* | 0.49±0.41 | 0.45±0.53 | 0.48±0.4 | 0.6331 | 1 | 0.5±0.5 | 0.53±0.38 | 0.44±0.38 | 0.5734 | 1 |
| *DKC1* | 35.23±24.15 | 36.31±20.9 | 33.25±20.48 | 0.7746 | 1 | 36.12±21.44 | 25.93±18.59 | 33.58±21.94 | 0.6118 | 1 |
| *GAR1* | 1.75±1.31 | 1.81±1.5 | 1.62±1.33 | 0.5357 | 1 | 1.79±1.46 | 2.49±0.83 | 1.61±1.33 | 0.1091 | 1 |
| *HMBOX1* | 0.56±0.64 | 0.74±1.17 | 0.47±0.47 | 0.5972 | 1 | 0.61±0.93 | 0.49±0.33 | 0.59±0.74 | 0.9163 | 1 |
| *MEN1* | 0.58±0.68 | 0.54±0.65 | 0.53±0.69 | 0.9872 | 1 | 0.49±0.51 | 0.41±0.25 | 0.63±0.83 | 0.814 | 1 |
| *NBS1* | 11.27±6.49 | 11.64±4.99 | 11.87±4.86 | 0.658 | 1 | 12.12±5.25 | 13.17±5.12 | 10.93±5.43 | 0.1836 | 1 |
| *NHP2* | 0.95±0.8 | 0.93±0.96 | 0.75±0.82 | 0.0725 | 1 | 0.92±0.96 | 0.88±0.52 | 0.81±0.76 | 0.5845 | 1 |
| *NME1* | 0.56±0.57 | 0.5±0.33 | 0.53±0.33 | 0.6815 | 1 | 0.51±0.35 | 0.71±0.47 | 0.54±0.45 | 0.5591 | 1 |
| *NOP10* | 0.45±0.34 | 0.4±0.53 | 0.41±0.36 | 0.0734 | 1 | 0.39±0.34 | 0.58±0.6 | 0.43±0.5 | 0.8405 | 1 |
| *OBFC1* | 0.58±0.78 | 0.38±0.49 | 0.43±0.55 | 0.494 | 1 | 0.41±0.58 | 0.48±0.28 | 0.49±0.63 | 0.5909 | 1 |
| *PARP1* | 0.66±0.39 | 0.77±0.97 | 0.56±0.27 | 0.2054 | 1 | 0.75±0.86 | 0.64±0.11 | 0.58±0.28 | 0.1497 | 1 |
| *POT1* | 0.63±0.73 | 0.39±0.35 | 0.48±0.44 | 0.2436 | 1 | 0.53±0.56 | 0.33±0.16 | 0.44±0.47 | 0.7227 | 1 |
| *RAD50* | 21.58±10.63 | 23.49±10.02 | 21.97±9.39 | 0.6735 | 1 | 22.62±10.63 | 21.53±8.57 | 22.36±9.27 | 0.8673 | 1 |
| *RAD51D* | 46.47±8.77 | 47.45±8.19 | 42.48±11.36 | **0.0077** | 0.2233 | 46.52±8.96 | 48.38±6.06 | 43.59±10.73 | 0.363 | 1 |
| *RAP1* | 0.59±0.44 | 0.49±0.28 | 0.55±0.4 | 0.8286 | 1 | 0.53±0.37 | 0.27±0.18 | 0.56±0.37 | 0.0783 | 1 |
| *RECQL5* | 0.52±0.5 | 0.87±1.65 | 0.67±0.64 | 0.4766 | 1 | 0.58±0.5 | 0.59±0.27 | 0.86±1.59 | 0.5416 | 1 |
| *RTEL* | 65.67±11.59 | 65.2±14.99 | 66.69±15.08 | 0.5914 | 1 | 67.11±13.01 | 66.43±11.94 | 64.26±15.74 | 0.5795 | 1 |
| *TCAB1* | 0.39±0.23 | 0.46±0.27 | 0.49±0.42 | 0.3683 | 1 | 0.47±0.37 | 0.51±0.21 | 0.42±0.28 | 0.5679 | 1 |
| *TEP* | 0.78±0.62 | 1.01±1.44 | 0.98±0.93 | 0.4374 | 1 | 0.81±0.96 | 0.72±0.38 | 1.12±1.25 | 0.103 | 1 |
| *TERC* | 1.41±2.5 | 1.33±1.97 | 1.01±2.58 | 0.3022 | 1 | 1.2±2.13 | 1.26±0.73 | 1.25±2.62 | 0.1646 | 1 |
| *TNKS1* | 0.48±0.52 | 0.52±0.85 | 0.55±0.7 | 0.4459 | 1 | 0.63±0.96 | 0.5±0.28 | 0.42±0.38 | 0.7008 | 1 |
| *TP53* | 0.51±0.39 | 0.77±0.68 | 0.7±0.76 | 0.1265 | 1 | 0.61±0.52 | 0.51±0.21 | 0.76±0.81 | 0.513 | 1 |
| *TPP1* | 0.68±0.66 | 0.64±0.99 | 0.59±0.43 | 0.5914 | 1 | 0.59±0.84 | 0.74±0.62 | 0.68±0.59 | 0.2523 | 1 |
| *TRF1* | 11.94±6.06 | 12.75±6.24 | 11.15±6.34 | 0.2442 | 1 | 12.35±5.98 | 16.44±6.94 | 11.28±6.41 | 0.0885 | 1 |
| 29 Genes | 8.24±1.26 | 8.32±1.14 | 8.06±1.42 | 0.6465 | / | 8.36±1.16 | 8.44±0.75 | 7.99±1.42 | 0.1391 | / |

^1^*P* values calculated with Kruskal-Wallis Rank Sum Test, ^2^ Holm's corrected *P* values, *P*<0.05 in bold
